# Does medical training in Thailand prepare doctors for work in community hospitals? An analysis of critical incidents

**DOI:** 10.1186/s12960-019-0399-8

**Published:** 2019-07-29

**Authors:** Dumrongrat Lertrattananon, Wirun Limsawart, Alan Dellow, Helen Pugsley

**Affiliations:** 1 Department of Family Medicine, Faculty of Medicine Ramathibodi Hospital, Mahidol University, Bangkok, Thailand; 2 Society and Health Institute, Ministry of Public Health, Nonthaburi, Thailand; 3 Oxford Deanery, Oxford, United Kingdom; 4 Centre for Medical Education, School of Medicine, Cardiff University, Cardiff, United Kingdom

## Abstract

**Background:**

Compulsory 3-year public service was implemented in 1967 as a measure to tackle the maldistribution of doctors in Thailand. Currently, therefore, most medical graduates work in rural community hospitals for their first jobs. This research explored doctors’ perceptions of preparedness for practice using a critical incident technique.

**Methods:**

A self-administered critical incident questionnaire was developed. Convenient samples were used, i.e. Family Medicine residents at Ramathibodi Hospital who had worked in a community hospital after graduation before returning to residency training. Participants were asked to write about two incidents that had occurred while working in a community hospital, one in which they felt the knowledge and skills obtained in medical school had prepared them for managing the situation effectively and the other in which they felt ill-prepared. Data were thematically analysed.

**Results:**

Fifty-six critical incidents were reported from 28 participants. There were representatives from both normal and rural tracks of undergraduate training and community hospitals of all sizes and all regions. Doctors felt well-prepared to provide care for patients in emergency situations and as in-patients, but under-prepared for obstetric and paediatric emergencies, out-patient care, and palliative care. Moreover, they felt poorly prepared to deal with difficult patients, hospital administration and quality assurance.

**Conclusions:**

Long-term solutions are needed to solve the rural doctor shortage. Medical graduates from both normal and rural tracks felt poorly prepared for working effectively in community hospitals. Medical training should prepare doctors for rural work, and they should be supported while in post.

**Keywords:**

Preparedness for practice, Community hospital, Critical incident, Rural doctor, Rural practice, Undergraduate medical training

## Background

Thailand, a South-East Asian country, has faced long-standing shortages of doctors in rural areas. In an attempt to tackle this problem, in 1967, the government enforced a 3-year period of public work for all doctors after graduation. This measure remains in place along with other strategies, including an increase in medical school places. However, working in rural areas is perceived by doctors as being a frightening experience, and a high resignation rate has been reported among newly graduated doctors under compulsory contract [1].

In Thailand, the undergraduate medical curriculum consists of a 6-year training programme typically divided into three phases, i.e. year 1 for general education, years 2–3 covering basic medical sciences, and years 4–6 for gaining clinical experience. There are two main tracks for medical training, “normal” and “rural”, differing in their admission process, place of training and place of work. Students from anywhere in the country can apply for normal track training through a central selection procedure. Their training is undertaken in traditional medical schools, and job placement is to any community hospital in the country. Students applying for the rural track are specifically selected from the provinces. Their training is undertaken in regional or provincial hospitals, and job placement is to community hospitals in the provinces or regions from which they come. The curriculum for both tracks is very similar, with clinical training usually taking place in tertiary or quaternary care hospitals and involving limited community experience. These learning environments may therefore fail to provide graduates with the competencies required for community hospital work.

Increasing the number of doctors in rural hospitals by training more doctors and imposing a 3-year contract on them to fill the shortage sounds appealing [2, 3]. However, the problem has proved to be too complex for a simple solution. Organisations tend to seek solutions that produce short-term improvements but create long-term side effects [4]. “Double-loop” thinking, involving deeper and more comprehensive enquiry, is called for. Solving the rural doctor shortage needs medical schools and government to learn from the consequences of previous actions before implementing new policies. Work in rural community hospitals is challenging for newly qualified doctors, yet little is known about their sense of preparedness for rural practice. Studying their perceptions can provide valuable feedback as well as potentially offering reasons for high resignation rates.

This research aims to explore doctors’ perceptions of their preparedness for working effectively in community hospitals in Thailand. This feedback should be of value to educators and policymakers responsible for the training and support of doctors, thereby contributing to the solution of the rural doctor shortage.

## Methods

Critical incident analysis evolved from military research but is now widely used in many disciplines [5, 6] including medicine [7–10] and healthcare quality research [11, 12]. It is also used frequently in medical education [13], for example to identify good professional practice [14], to elicit necessary competencies [15, 16], to evaluate programmes [17], to explore preparedness for practice [18] and to promote reflective learning [19]. This approach facilitates objective inference from direct experience or direct observation of an event and has been proven to have both reliability and validity [20, 21]. The procedure is also flexible, modifiable and efficient due to the use of extreme cases only [5].

A self-administered critical incident questionnaire was developed and piloted among 10 health professionals to ensure that the questions were readily understandable and were likely to draw relevant answers. The questions were as follows: “Thinking back to your early days when you finished medical school and started working in a community hospital as a doctor, can you think of an experience of a patient encounter which you felt well-prepared for by your medical school in order to manage the patient effectively”. “Describe what happened”. “Who was involved (no names)”. “Give details of the experience”. “What and why did you feel well-prepared?” “Thinking back to your days in medical school was there anything in particular that you learnt or did that helped you feel well-prepared when dealing with this experience”. “And how did things turn out?” The same questions were then posed again but asking participants to relate these to critical incidents they felt they were NOT well prepared for.

For the convenience of sampling, the participants were Family Medicine residents at Ramathibodi Hospital. Of these, 17 had undertaken their training through a rural track programme and 11 through a normal track. Residents who had not worked in a community hospital were excluded. All participants gave informed consent before being interviewed. A group interview using the self-administered critical incident questionnaire was conducted on 22 June 2018: 17 rural and 11 normal.

Data were analysed using thematic analysis [22]. First-round coding was undertaken by identifying the keywords. The first-round codes were subsequently grouped into themes and sub-themes. The provisional themes and sub-themes were reviewed and revised by two researchers (DL and WL) and were categorised and named against the Thai Medical Council’s Professional Standards 2012. Final themes were discussed and agreed to ensure due process was adhered to.

This study and its protocol received full ethical approval from the Postgraduate Medical and Dental Education Ethics Committee at Cardiff University, Wales, United Kingdom.

## Results

Fifty-six critical incidents were reported from 28 first to third-year Family Medicine residents. Eleven participants were trained in a typical medical programme, and 17 were on the rural track. The characteristics of the participants are shown in Table 1.

**Table 1:** Characteristics of participants

No.	Gender	Training			Working in community hospital			Themes*	
		Graduation year	Location	Track	Duration (months)	Region	Size	Well-prepared	Not well-prepared
1	F	2013	Other	Rural	15	N	F3	3.2	3.1 (paediatrics)
2	F	2012	Other	Rural	27	W	F3	3.1	3.2
3	F	2013	Other	Rural	14	NE	F3	3.1	1.3
4	M	2014	Other	Rural	27	S	F2	2.1	1.1
5	F	2013	Other	Rural	26	N	F2	1.2	4
6	M	2013	Bangkok	Normal	39	E	F2	3.1	3.1 (obstetrics)
7	F	2014	Bangkok	Normal	3	C	F2	3.1	2.1
8	M	2014	Bangkok	Normal	27	NE	F2	3.1	3.1 (paediatrics)
9	F	2010	Other	Rural	63	NE	F3	3.1	3.1 (obstetrics)
10	M	2015	Other	Rural	2	S	F1	3.1	3.2
11	F	2012	Other	Normal	27	NE	F3	3.1	3.2
12	F	2014	Bangkok	Normal	26	NE	F3	3.1 (except procedural skills)	3.1 (adult and obstetric procedural skills), 3.2
13	F	2013	Bangkok	Normal	37	C	F2	3.2	4
14	M	2014	Other	Rural	26	N	F2	3.1	1.1
15	M	2013	Other	Rural	40	E	F2	3.2 (IPD)	3.2 (OPD)
16	M	2011	Other	Rural	3	N	F2	1.2	3.1
17	F	2013	other	Rural	39	N	F2	3.1	3.2
18	F	2014	Bangkok	Normal	28	NE	F2	3.2 (IPD)	3.2 (OPD), 4
19	F	2014	Other	Rural	26	N	F3	1.2	3.2
20	F	2012	Other	Rural	44	C	F2	3.1	3.1 (ultrasound)
21	M	2014	Other	Rural	3	W	F3	3.1	2.2
22	F	2012	Bangkok	Normal	27	N	F3	1.2	1.1
23	F	2000	Bangkok	Normal	180	S	F3	3.1	1.1
24	M	2016	Other	Rural	3	C	M2	2.1	3.1
25	F	2016	Bangkok	Normal	3	C	F3	3.1	2.2, 3.2
26	F	2014	Other	Rural	26	S	F3	2.2, 3.1	3.1 (paediatrics)
27	F	2014	Other	Rural	26	N	F2	3.1	3.2
28	F	2014	Bangkok	Normal	25	W	F2	3.1	1.1

*N* north, *NE* northeast, *E* east, *W* west, *S* south, *C* central, *F3* 30 bed-sized community hospital, *F2* 30–90 bed-sized community hospital, *F1* 90–120 bed-sized community hospital, *M2* ≥ 120 bed-sized community hospital

*Please refer to Table 2 for the meaning of the themes

We identified three themes from the critical incident reports: perceptions of preparedness for rural practice, effective educational methods and discrepancies between training and real practice. For perceptions of preparedness for rural practice, we identified four major sub-themes and named these using the Thai Medical Council’s Professional Standards 2012: Communication and interpersonal skills, Scientific knowledge of medicine, Patient care, and Administration and quality assurance. The sub-themes of perceptions of preparedness for rural practice are shown in Table 2.

**Table 2:** Perceptions of preparedness for rural practice

No.	Sub-themes	Definitions	Examples	Well-prepared*	Not well-prepared*
1	Communication and interpersonal skills				
1.1	Difficult patients	Communication and interpersonal skills with specific types of difficult patients	Anger, a very important person, drunk, intimidate	–	N4, N14, N22, N23, N28
1.2	General patients	Communication and interpersonal skills between doctor and patients/relatives	Building relationship, conciliation, revealing medical error	W5, W16, W19, W22	–
1.3	Colleagues	Communication and interpersonal skills between colleagues	Understanding other people	–	N3
2	Scientific knowledge of medicine				
2.1	Family medicine	Knowledge of family medicine approaches	Holistic care, continuity of care, explore concern and expectation, empathy	W4, W24	N7
2.2	Medical laws and forensic medicine	Knowledge of medical laws and forensic medicine	Post-mortem examination, medical certificate, certificate for disability	W26	N21, N25
3	Patient care				
3.1	Emergency conditions	Clinical knowledge and skills in history taking, physical examination, investigation, differential diagnosis, management, referral, managerial skills	Life-threatening conditions—primary survey and life support in trauma and cardiac arrest (BLS, ACLS, ATLS)	W2, W6, W17, W20, W21, W26	–
			Medical or surgical emergency and procedures—sepsis, arrhythmia, anaphylaxis, gastrointestinal haemorrhage/perforation, intubation, intercostal drainage, etc.	W7, W8, W9, W10, W11, W12 (except procedural skills), W14, W23 (appendectomy), W25, W27, W28	N16 (referral problem) N20 (ultrasound) N24 (ECG reading)
			Paediatric emergency and procedures	–	N1 (infant intubation) N8 (umbilical venous catheterisation) N26 (supraventricular tachycardia)
			Obstetrics emergency and procedures	W3 (assessment of labour) W23 (caesarean section, assisted breech delivery, vacuum extraction) W26 (manual removal of the placenta)	N6 (manoeuvres for shoulder dystocia) N9 (tubal resection) N12 (normal delivery)
3.2	Non-emergency conditions	Clinical knowledge and skills in history taking, physical examination, investigation, differential diagnosis, management, referral, managerial skills	Out-patient department (OPD)—non-communicable diseases, chronic multiple illnesses, tuberculosis, chronic kidney disease, eye diseases	W1, W13	N2, N10, N12, N15, N18, N25, N27
			In-patient department (IPD)—ward round	W15, W18	–
			Palliative care	–	N17, N19
			Paediatrics	W27 (newborn)	N11 (psychiatry)
4	Administration and quality assurance	Knowledge of hospital administration, hospital accreditation	Government rules and regulations, hospital accreditation (HA)	–	N5, N13, N18

The number after W and N means an identification number of a participant; please see characteristics of participants in Table 1

*W* well-prepared, *N* not well-prepared

The analysis did not show differences in preparedness for practice regarding size or region of hospitals and the undergraduate tracks.

### Perceptions of preparedness for rural practice

#### Communication and interpersonal skills

Ten incidents were related to communication and interprofessional skills. Four participants felt well-prepared in these skills. However, the remaining five participants felt they were not well-prepared to deal with difficult and challenging patients, in particular, angry and demanding patients. Another doctor reported that she felt she was treated unfairly by her colleagues and she lacked skills to understand them.

A member of staff in our hospital asked me to admit his 10-years-old boy who had fever, abdominal pain, nausea, vomiting and diarrhoea. I diagnosed him with viral gastroenteritis and gave him supportive treatment. The boy’s abdominal pain didn’t improve and they wanted to be referred. I was also very stressed because I couldn’t make them understand or relieve their concern, so I had to unnecessarily refer the patient to a provincial hospital. (N14, male, rural track)

The communication skills of physicians have been shown to be linked with patients’ perceptions of their competence and the incidence of malpractice claims [23]. Furthermore, communication skills training can improve patient satisfaction [24, 25] and affect levels of empathy and burnout among physicians [25]. Teaching effective communication skills is therefore a vital part of training and can be undertaken using role-play, feedback and debriefing [26].

### Scientific knowledge of medicine

#### Family medicine

Knowledge of a family medicine approach was mentioned by three participants. Two of these reported being able to apply family medicine concepts such as practising holistically, providing continuity of care, and exploring patients’ concerns (W4, male, rural track, and W24, male, rural track). The other participant felt she was not well-prepared to provide holistic care and could not listen to patients properly or explore their illness (N7, female, normal track).

#### Medical laws and forensic medicine

Knowledge of medical law and forensic medicine was mentioned by three participants, two of whom felt under-prepared. One reported that she did not remember how to write a certificate of disability (N25, female, normal track), and one reported that he was worried that his opinion about a wound might affect a medico-legal trial (N21, male, rural track). One participant felt well-prepared to perform post-mortem examinations because she had been trained in a regional hospital in the south of Thailand where there has been civil unrest and violence for a long time (W26, female, rural track).

I was worried about my opinion given to the police because it was several months from the examination date and I found that I didn’t write a lot in the medical record. I was worried about a lawsuit. … I hadn’t experienced this situation during my medical training and there was little teaching about this. (N21, male, rural track)

### Patient care

#### Emergency conditions

Seventeen doctors from both training tracks reported feeling well-prepared to provide care to adult patients with life-threatening and emergency conditions, including performing necessary procedures in such situations. However, one doctor mentioned that if she had learned to assess the inferior vena cava by ultrasound, she could have assessed the patient’s volume status more effectively during resuscitation (N20, female, rural track). Another doctor reported that he had misinterpreted an electrocardiography (ECG) of ST-segment elevation myocardial infarction (STEMI) as being non-STEMI which could have caused a delay in fast-tracking the patient (N24, male, rural track).

A 40-year-old man was delivered to ER (emergency room) after a motorcycle accident. Physical examination revealed decreased breath sounds over the left lung and there was a stepping of ribs. I didn’t hesitate to put an ICD (intercostal drainage) into his chest immediately rather than sending him for chest x-ray which was far away and would take more time and would not have saved his life. (W20, female, rural track)

Even though doctors felt quite well-prepared to deal with emergency situations, they found this stressful because the medical system was unsupportive. One doctor reported that he was refused by three larger hospitals when trying to refer a patient with active upper gastrointestinal haemorrhage. Finally, he resigned from his government contract.

Medical School only taught me best practice in CPG (clinical practice guideline) “on paper”, but what if I couldn’t do it in a real life situation. I didn’t learn what to do if I was unable to refer an emergency patient. …When I finished the intern year, I resigned. I didn’t dare to work there anymore. What a stressful and risky system! (N16, male, rural track)

One doctor who graduated in 2000 felt well-prepared for many surgical and obstetric procedures (W23, female, normal track). She reported having performed many appendectomies and caesarean sections in her community hospital and felt well-prepared to undertake vacuum extractions and assisted breech deliveries. Most community hospitals have now closed their operation rooms to avoid medico-legal risk taking with the result that some surgical procedures, such as appendectomy and caesarean section, are no longer required competencies for newly graduated doctors. However, critical incident reporting showed that doctors in community hospitals are still required to undertake many obstetric procedures, such as normal delivery, tubal resection, manoeuvres for shoulder dystocia, and manual removal of placenta. Many doctors felt under-prepared for these as well as paediatric emergencies and procedures.

A pregnant Cambodian woman, G2P1, no ANC (antenatal care), came to ER with labour pain and broken waters. .. After examination I told her that the baby was estimated to be 4,000 grams and I would refer her for delivery at the regional hospital because the baby was big. She insisted on being delivered in the community hospital. She had no medical benefit and no money and said she was able to deliver her first baby who was 4,500 grams. … Thirty minutes later, she had more pain and started to go into labour. After delivery of the head, the shoulder would not pass. I called the director for help. He helped deliver the baby successfully, but the baby had asphyxia and cardiac arrest. After 30 minutes of CPR (cardiopulmonary resuscitation) we couldn’t save the baby. The mother was sad but accepted the situation and didn’t want to sue. But I felt guilty and scared every time there was a woman in labour under my charge. (N6, male, normal track)

A 2 months old infant came to ER with fever, cough and dyspnoea. She needed intubation but I didn’t feel confident that I could do it. I made one attempt but failed. My friend came to help and successfully intubated the baby after 2 attempts. … I had never done this before in Medical School. (N1, female, rural track)

#### Non-emergency conditions

Ten participants from both training tracks felt poorly prepared to manage non-emergency conditions, such as common and chronic diseases seen in OPD, child mental health, and palliative care. Three doctors who had graduated from established medical schools in Bangkok reported that they benefited from the strong academic environment. However, two of them reported being able to diagnose rare diseases, but did not confidently treat common diseases.

A child was brought to the hospital by his granny being hyperactive, very naughty, and with delayed speech. …. I didn’t know how to deal with this running-around boy. ….I had seen very few cases in paediatric psychiatry. (N11, female, normal track)

The patient was referred back for palliative care. He developed more pain and frequently came to ER for an extra shot of morphine. At that time, I didn’t know about palliative care and pain control. Even though the dose of morphine was very high, it still could not control his pain. He still suffered from pain until he died. If I had known about pain control then, I would have helped him pass away peacefully. (N17, female, rural track)

### Administration and quality assurance

In addition to clinical practice, working effectively in community hospitals requires doctors to have the knowledge and skills necessary for hospital administration and quality assurance. Three participants mentioned these competencies, and each of them felt under-prepared.

I was assigned HA job and the chairman of PCT (Patient Care Team). I didn’t know anything about HA and no one was able to explain me clearly what I had to do to. … In medical school, I only learned about diseases and patient care. I’d never learned about bureaucracy system in government hospitals and hospital accreditation. (N18, female, normal track)

### Effective educational methods

Lectures, observation or being an assistant were felt to be insufficient as forms of training for procedural skills. While in training, having an opportunity to practise in the “real world” and with a sufficient number of patients made participants feel well-prepared. However, busy out-patient departments were felt to offer few opportunities for teaching and learning [27].

Many participants felt they were well-prepared for resuscitation skills because these are taught extensively and seen as a priority in medical schools. Participants also identified factors that made learning more effective, for example small group teaching, close supervision, being given feedback, repetition, receiving advice and tips on techniques, and dividing complex task into more manageable steps. In addition, many highlighted the idea that examinations motivated them to learn, reinforcing documented findings [28–30].

### Discrepancies between training and real practice

Participants reported a mismatch between training activities and their actual work. During training, most of their time was spent on wards but in post most of their work took place in OPD. Similarly, many of the cases seen in medical school were complex and seen only rarely in community hospitals. As a consequence, they felt ill-prepared to deal with many of the more common conditions presenting to them. Also, there were differences between the treatment and management of patients in medical schools and community hospitals. For example, in medical school hospitals, obstetric mal-presentations usually proceeded to caesarean section whereas in community hospitals it was often necessary to perform assisted breech delivery, vacuum extraction and other manoeuvres as prompt referral to surgical centres was impossible. This highlights the need for medical training to be relevant to meeting the healthcare needs of the population [31].

## Discussion

This study has demonstrated that critical incident analysis can help provide a picture of perceptions of preparedness for practice. It also gave valuable information about the context within which competencies are practised as well as highlighting those that are particularly important and relevant. This confirmed validity of the technique and its utilisation as the previous study has done [17]. Recall bias could be a potential limitation of the study, as participants were asked to think about critical incidents which might have occurred several years previously. However, the critical incident technique asks participants to recall their most positive and negative events, which are likely to be memorable.

The transition from medical school to clinical practice is considered to be a stressful change, due to an increase in responsibility, workload, interaction with patients, and involvement in multi-professional health care teams [32, 33]. Managing uncertainty and feeling unsupported increase stress levels during this transition period [33].

The competencies required for rural practice differ from those of general practitioners working in urban areas [34–36]. In addition to out-patient practice, doctors in community hospitals also need to be able to manage life-threatening and emergency conditions, provide in-patient care and perform minor surgery and obstetric procedures. In this study, areas of practice in which participants felt under-prepared resembled those selected as “under-trained” in a satisfaction survey of medical graduates [37]. Graduates felt under-trained in clinical, technical and procedural skills, though this was less marked for procedures in internal medicine than in obstetric and surgical procedures [37].

Adequate experiential learning was associated with a perception of being well-prepared for practice [38, 39]. Also, clinical placements should support opportunistic learning [40] and include students in teamwork by giving them more responsibility in patient care [39, 41]. In Thailand, the final year of undergraduate medical training (year 6) is based on an apprenticeship model during which teaching and learning are workplace-based [42]. This is important for acquiring skills that can only be learned through practical experience, such as clinical prioritisation, time management, and dealing with paperwork [39]. However, hospital-based education provides students with a narrow view of health problems [40]. To better prepare medical graduates for rural practice, it would appear to be obvious that they should be exposed to learning experiences in rural areas [38, 43, 44].

## Limitations

The study results are not generalisable because of the small sample size and the fact that participants were doctors who had chosen to train on a postgraduate Family Medicine residency programme. This could mean that they share similar characteristics, such as recognition of the importance of communication skills with difficult patients.

This study did not explore the background of participants, whether urban or rural, nor the non-clinical aspects of preparedness for rural practice. Studies have shown that doctors with a rural background, as well as urban doctors who have undertaken a rural family medicine rotation, report feeling prepared for non-clinical aspects of rural practice, such as time management, understanding rural culture and living within a small community [45].

## Conclusions

This small-scale study is an exploratory investigation of doctors’ perceptions of preparedness for practice in community hospitals using a critical incident technique. Medical graduates from both normal and rural tracks shared similar perceptions of under-preparedness for working effectively in community hospitals. This has significant implications for curriculum development and the formulation of long-term solutions to the rural doctor shortage. A more extensive study is needed to obtain a country-wide view, but our findings reveal a discrepancy between medical school teaching and the competencies required by doctors working in community hospitals. Teaching communication and procedural skills should be prioritised and a much greater proportion of undergraduate training should be community-based. Community hospital doctors provide a vital service and as such deserve adequate preparation, a safe working environment, and adequate support. Their patients deserve no less than this.

## Data Availability

The Governance and Compliance Framework of Cardiff University requires all non-clinical research data generated by staff and/or postgraduate research projects to be stored securely by the academic unit for a period of no less than 5 years and at least 2 years post publication. Please contact the author for data requests.

## Abbreviations

ACLS: Advanced Cardiovascular Life Support
ANC: Antenatal care
ATLS: Advanced Trauma Life Support
BLS: Basic Life Support
CPG: Clinical practice guideline
CPR: Cardiopulmonary resuscitation
ECG: Electrocardiography
ER: Emergency room
G2P1: Gravida 2 para 1
HA: Hospital accreditation
ICD: Intercostal drainage
IPD: In-patient department
OPD: Out-patient department
PCT: Patient Care Team
STEMI: ST segment elevation myocardial infarction
